# Identification of Water Bodies in a Landsat 8 OLI Image Using a J48 Decision Tree

**DOI:** 10.3390/s16071075

**Published:** 2016-07-12

**Authors:** Tri Dev Acharya, Dong Ha Lee, In Tae Yang, Jae Kang Lee

**Affiliations:** 1 Department of Civil Engineering, Kangwon National University, Chuncheon 200-701, Korea; tridevacharya@gmail.com (T.D.A.); intae@kangwon.ac.kr (I.T.Y.); 2 LX Korea Cadastral Surveying Corporation, 141 Uisadang-daero Yeodeungpo-gu, Seoul 150-911, Korea; jaekang.lee@lx.or.kr

**Keywords:** Landsat 8, OLI sensor, J48 decision tree, water body identification, Gandwon-do

## Abstract

Water bodies are essential to humans and other forms of life. Identification of water bodies can be useful in various ways, including estimation of water availability, demarcation of flooded regions, change detection, and so on. In past decades, Landsat satellite sensors have been used for land use classification and water body identification. Due to the introduction of a New Operational Land Imager (OLI) sensor on Landsat 8 with a high spectral resolution and improved signal-to-noise ratio, the quality of imagery sensed by Landsat 8 has improved, enabling better characterization of land cover and increased data size. Therefore, it is necessary to explore the most appropriate and practical water identification methods that take advantage of the improved image quality and use the fewest inputs based on the original OLI bands. The objective of the study is to explore the potential of a J48 decision tree (JDT) in identifying water bodies using reflectance bands from Landsat 8 OLI imagery. J48 is an open-source decision tree. The test site for the study is in the Northern Han River Basin, which is located in Gangwon province, Korea. Training data with individual bands were used to develop the JDT model and later applied to the whole study area. The performance of the model was statistically analysed using the kappa statistic and area under the curve (AUC). The results were compared with five other known water identification methods using a confusion matrix and related statistics. Almost all the methods showed high accuracy, and the JDT was successfully applied to the OLI image using only four bands, where the new additional deep blue band of OLI was found to have the third highest information gain. Thus, the JDT can be a good method for water body identification based on images with improved resolution and increased size.

## 1. Introduction

Water is an essential component of ecosystems for the sustainability of life on earth. It balances ecosystems and maintains climate variation, carbon cycling, etc. It is equally important to humans and other forms of life. Its presence causes increases in forest and grassland, or vice versa, whereas its excess or absence could lead to disasters and extreme land use change. Hence, identification of water bodies is an essential process in science and engineering research. The identification can be useful in various ways, such as estimation of water areas [1,2], demarcation of flooded regions [3,4], wetland inventories [5,6], change detection [7,8], and so on. The availability of water helps in the estimation of agricultural land irrigation, productivity, hydropower energy, and many others. Floodplain area demarcation can be essential in land use planning and prevent damage in the future. Similarly, wetland inventories are essential for understanding wetland availability, ground water recharge estimations, etc. Changes in water surfaces using multi-temporal images can be performed. Moreover, water quality can also be assessed by remote sensing after the identification of water bodies [9]. The potential is vast, but only if the proper identification of water bodies can be improved.

Landsat series have been widely used in the identification of water bodies [1,2,7,8,10,11,12,13,14,15,16]. With the launch of Landsat 8 [17] on 11 February 2013, the improved Operational Land Imager (OLI) sensor and Thermal Infrared Sensor (TIRS) were introduced. The OLI captures images in nine spectral bands, whereas TIRS uses two. The differences between Landsat 8 bands and the previous Landsat 7 and Landsat 5 are shown in Table 1. Landsat 8 offers scientists a clearer view, with better spatial resolution than most ocean-sensing instruments, and greater sensitivity to brightness and colour than previous Landsat versions. It has two additional bands: a deep blue band (band 1) and a cirrus band (band 9). The deep blue band is for improved sensitivity to chlorophyll and other suspended materials in coastal waters and for retrieving atmospheric aerosol properties. The cirrus band is for cirrus cloud detection. Other typical bands are much narrower, whereas thermal bands are divided into two. A quality assurance band is also included to indicate the presence of terrain shadowing, data artefacts and clouds. Both sensors provide improved signal-to-noise ratio (SNR) radiometric performance quantized over a 12-bit dynamic range [18,19]. The improved OLI 12-bit radiometric resolution will enable better characterization of land cover states and conditions, particularly over water [20]. High SNR values are very important for identification of water bodies because very low signals from water cause variations in water quality to be lost in the noise of low SNR systems [21]. Landsat TM and ETM+ images have limited capability in the cases of freshwater and coastal waters [22,23]. These limitations are due to the relatively low SNR, as well as the limited number of spectral bands in the visible region where water quality spectral signatures are apparent [20]. Additionally, due to the increase in quality, the image size also increases. The average scene size of previous Landsat series i.e., Landsat 5 and 7, are 263 MB and 487 MB, respectively, whereas Landsat 8 average scene size has increased to 1813 Mb (approximately 1 GB compressed, and 2 GB uncompressed) [19,24]. Thus, it is necessary to explore the efficiencies of various water identification techniques for images with improved radiometric resolution and increased data size, such as OLI images.

In past years, several contributions had been made toward the identification of water bodies from remotely sensed images [7,10,12,15,16,25,26,27,28,29,30,31,32,33]. However, it remains a challenge due to factors such as complexity of the landscape in a study area, selected remotely-sensed data, and classification methods [34]. Water body identification methods can be categorized as follows: (a) digitizing through visual interpretation, which is highly accurate, but labour intensive; (b) density slicing of a single band [11,35,36,37], which applies a fixed threshold in a given spectral band [8]; (c) calculating spectral indices [10,12,30,38,39], which combines two or more bands by mathematic ratios; and (d) classification of multispectral data using unsupervised [40] and supervised techniques [41,42,43,44].

Due to simplicity, low cost, and superior performance based on specific noises, water indices are widely used for identification of water bodies [45]. Some of the most well-known multiband water indices’ methods include the Normalized Difference Water Index (NDWI) [10], Modified NDWI (MNDWI) [12], and Automated Water Extraction Index (AWEI) [30]. NDWI was developed to identify water surfaces from Landsat images. It uses green and near-infrared (NIR) bands to maximize water body identification, but it has errors over built-up lands. Using mid-infrared (MIR), MNDWI can overcome NDWI problems by removing built-up lands and soil noises. Similarly, AWEI was proposed with (AWEI_sh_) and without shadows (AWEI_nsh_) to identify water bodies [32]. Water bodies are identified by positive values in all of these indices [7]. Classification methods use statistical pattern recognition techniques to extract surface water [29]. These methods are more accurate than others, as they do not need to set empirical thresholds [8]. They can be unsupervised or supervised. Unsupervised methods use clustering or region growing, whereas supervised techniques require inputs from users to develop training rules that apply over whole regions. Well-known supervised methods include maximum likelihood (ML) [11], decision trees [33,41,43,44,46], artificial neural networks (ANN) [47], and support vector machines (SVM) [42,48]. For further information, Karpatne et al. [49] provide a comprehensive review of remote sensing-based identification methods of inland water bodies.

The decision tree method has been widely used in small- [27] to large-scale study areas [41,46]. Most existing applications of decision trees use additional explanatory variables, e.g., indices, slope, and hill shade from original bands [27,41,46]. These additional variables require extra efforts to create and increase the data size and computing efforts. With the improved radiometric properties and increased data size of Landsat OLI, there is a need to explore the most appropriate and practical water identification methods that take advantage of improved image quality and use minimal data, i.e., the minimum number of bands. Hence, in this study, the main objective is to identify water bodies in Gangwon province in Landsat 8 OLI image using a J48 Decision Tree (JDT). Note that we consider only original OLI reflectance bands as input data for water and non-water classification. In this study, we selected a suitable test site, choose one OLI image and sampled training and validation data. Apart from the JDT, five other water indices were used to identify water bodies for comparison: density slicing, NDWI, MNDWI, ML, and SVM. This study explores the potential of the JDT for water body identification using satellite image original reflectance bands with improved radiometric resolution and increased data size.

## 2. Materials and Methods

**Figure 1:**
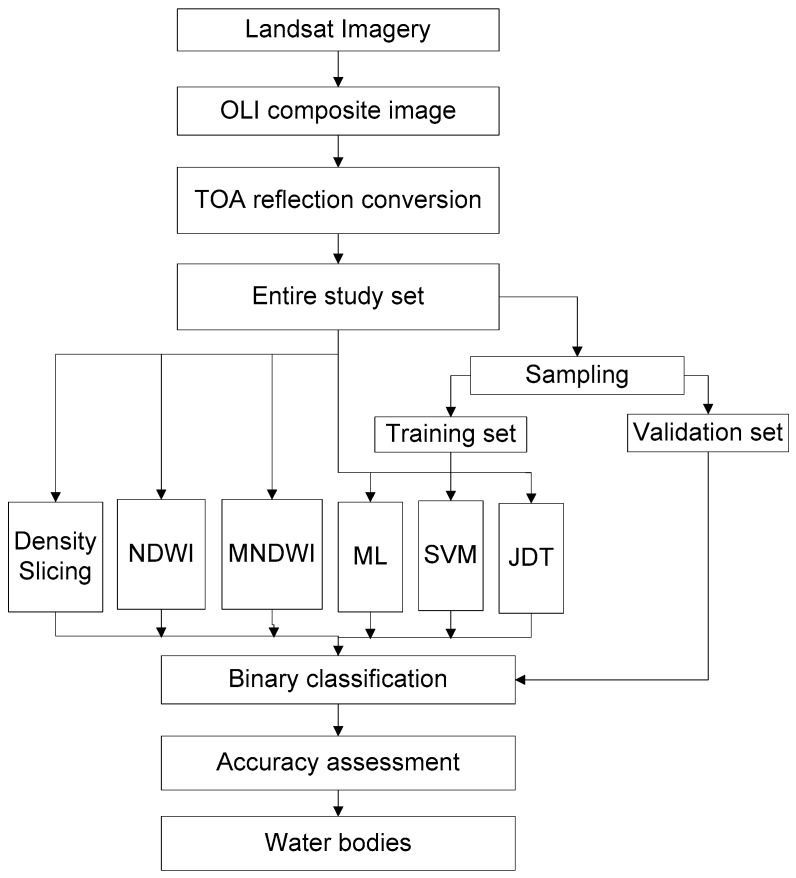
Overall flowchart adopted in this study.

To fulfil the objectives of the study, a suitable test site was selected. After obtaining the data, it was calibrated and pre-processed. Sampling for training, validation, and binary water and non-water classification was then performed. Figure 1 shows the overall method adopted for identification of water bodies using the JDT in this study.

### 2.1. Test Site

**Figure 2:**
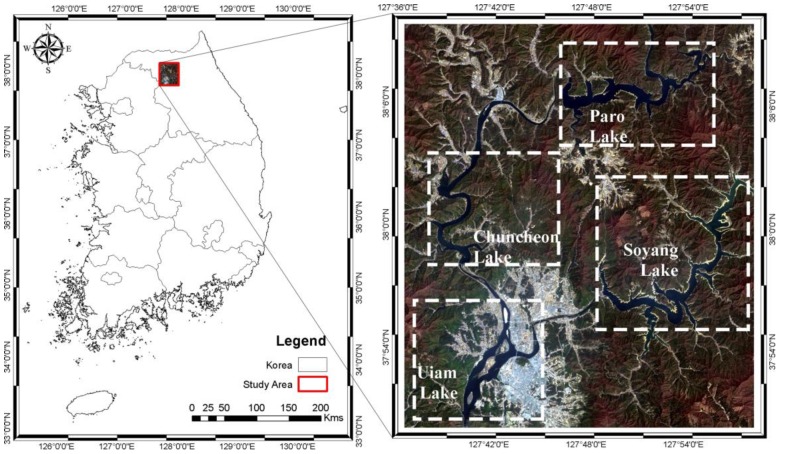
Location of the test site in Korea, with lakes shown in a Landsat 8 natural colour composite image taken from 11 February 2013. Each box includes the lake name.

The Northern Han River flows from Gangwon and through Gyeonggi province in the Republic of Korea. A rectangular area in the Northern Han River Basin, located in Gangwon province, was selected as the test site (Figure 2). It is located between 37°50′4.723′′–38°10′1.437′′ N and 127°36′47.037′′–127°58′3.853′′ E. In addition to water, the land cover types at the site are urban, forest, vegetation, and soil. The terrain is complicated, with hilly and plain areas. It consists of four artificial lakes formed by dams in the river: Paro Lake, Soyang Lake, Chuncheon Lake, and Uiam Lake.

Built in May 1944 with the construction of the Hwacheon Dam in Japan, Paro Lake is a reservoir in the valley of the Northern Han River. The dam sits at the head of a 3901 square kilometre catchment area, and the reservoir has a gross capacity of one billion cubic metres. Of this capacity, 809 million cubic metres can be regulated and 213 million cubic metres is used for flood control. The reservoir surface area is 38.9 square kilometres. At the time of the Korean War, it was one of South Korea’s only sources of power. It is still one of Korea’s largest reservoirs and an important source of electrical power, with a capacity of 108,000 kW. Similarly, built in February 1965, Chuncheon Lake is a reservoir at the foot of Chuncheon Dam in the upper region of the North Han River. It was formed by blocking the flow of water from Paro Lake. Its length is approximately 21.8 km. It is also a hydropower reservoir, with a capacity of 62,280 kW. Soyang Lake, built in 1973, is a reservoir formed by an embankment dam on the Soyang River. Its gross water storage capacity is 2.9 billion tons, the second largest in Korea after Chungju Dam (2.75 billion tons). The purposes of the dam are flood control, water supply and hydroelectric power generation. The 123 m tall dam withholds a reservoir of 29 billion cubic metres and supplies water to a 200,000 kW power station. Uiam Lake was formed by a dam built on the North Han River in 1967. It is a reservoir for a hydropower plant with a capacity of 46,500 kW. The lake extends upstream to Soyang Dam and Chuncheon Dam, which are located at distances of 19.8 and 17.5 km, respectively. It also has three small islands inside.

In Korea, the selected site is the only place that contains four large, clear freshwater bodies in such a small area. Thus, it is a suitable site for testing water body identification algorithms. The water in these lakes changes seasonally, especially in Soyang and Paro during the winter. However, over short periods, e.g., one season, the water is predominantly static, and the reservoirs store maximum amounts of water.

### 2.2. Data

The Level 1 Terrain-Corrected data acquired by the Landsat 8 OLI sensor on 24 April 2015 were collected from the United States Geological Survey (USGS) Global Visualization Viewer (GLOVIS) portal. The obtained multiband image coastal blue, blue, green, red, near-infrared (NIR), shortwave infrared 1 (SWIR_1), and shortwave infrared 2 (SWIR_2) bands (Table 2) were converted to top-of-atmosphere reflectance using the Landsat calibration tool in ENVI 5.1. The required coefficients and values, including the data acquisition date and sun elevation, were obtained from the Landsat MTL header file.

A 30-metre resolution scene of 1216 rows and 1054 columns was extracted for the study. Each of the pixels with all band values was exported into a comma-separated value (CSV) table for classification. Out of the whole scene, stratified random pixels were sampled for training and the validation of model development.

### 2.3. Methods

The J48 model was used in the Waikato Environment for Knowledge Analysis (WEKA) data mining environment [50]. JDT is an open source Java implementation of the C4.5 [51] decision tree implemented using the WEKA tool. Classification of a new item in the algorithm first requires a decision tree based on the attribute values of the available training data. Based on the available set of items in the training data, it identifies the attribute that classifies the various instances most clearly. The feature that tells us most about the data instances, i.e., which could lead to best classification, is said to have the highest information gain. Based on possible values of the feature, branches are terminated, and a target value is assigned. In other cases, the algorithm searches for other attributes that give us the highest information gain. The process continues in this manner until a clear decision regarding the combination of attributes that gives us a particular rule for determination of a target value is achieved. With the help of this decision tree, all of the respective attributes and their values undergo checking, thereby assigning or predicting the target values of all new instances. The decision tree method exhibits high accuracy across many environments, allocating more homogenous datasets based on binary splits [46]. These binary splitting nodes are based on conditions of explanatory variables that can be easily understood [33] and implemented in GIS.

From the entire study area scene, 7070 (70%) pixels were selected for training and 3065 (30%) pixels were selected for validation, such that both sets contained 50% of each water and non-water class. The sampled points were assigned water (class 1) and non-water (class 0) classes based on two methods. The main water bodies, which are well banked and remain relatively static throughout the year, were based on the water bodies layer of the digital topographic map version 2.0 provided by the Korean National Geographical Information Institute. The digital map was updated on 13 December 2015 using 25 cm resolution aerial photographs and field verification, which accurately represents the water bodies. For smaller water bodies and confusing island and land-water boundaries, expert’s opinions were used to check and re-assign the class. During the process, extra pixels were also added manually to ensure small water bodies were included. JDT was used to develop the model using a training dataset. All of the processing was executed using the default parameters in WEKA. The model was cross-validated using the remaining 30% of data for error estimation. The performance of the results was statically analysed using the kappa statistic and area under the curve. The kappa statistic measures the agreement between a prediction and the true class, where 1.0 signifies complete agreement. The area under the curve is widely used to measure the performance of a binary classifier, where a value of 1.0 represents a perfect test and 0.5 is a non-meaningful test [52]. Then, the models that provided splitting conditions were applied to the whole scene to perform binary water and non-water classification.

In addition to the JDT, five well-known water identification techniques were also implemented in the study area to compare the results. Density slicing of the SWIR_1 band was performed with a threshold value of 0.063 manually. Since it penetrates only a short distance into water, where it is absorbed with very little reflection, surface water features have very dark tones [11]. Both NDWI and MNDWI were calculated using spectral index methods and used for the identification of water. The calculations of these indices are shown in Table 3. Similarly, ML and SVM were used for the identification of water bodies in the same study area for comparison. ML is, by far, the most used classification method, whereas SVM is one of the most important state-of-the-art classification methods. The final results were compared using a confusion matrix and related statistics (e.g., overall accuracy, kappa coefficient, and the user’s and producer’s accuracy of each category).

Table 4 shows a typical layout of an m × m error matrix, where m is the number of classes, with the columns representing the reference data and rows representing the classified values, although both can be interchanged. In the table, diagonal elements are the pixels of agreements, whereas off-diagonal elements are disagreements. The accuracy of classification can be interpreted easily using the method of percentage of pixels correctly allocated, i.e., the overall accuracy of the classification. The individual class accuracy is the percentage of correctly-allocated pixels in a class to the total number of pixels in that class. They are called user’s accuracy and producer’s accuracy based on the matrix column and row allocation. However, these do not take into account the agreements between datasets that are due to chance alone. Hence, the kappa coefficient of agreement has been often used. It is a measure of agreement based on the difference between the actual agreement in the error matrix and the chance agreement. The calculations of these accuracies and the kappa coefficient are as follows:

(1) $$\text{User}’\text{s}\;\text{accuracy}=\frac{n_{\text{kk}}}{n_{k+}}$$

(2) $$\text{Producer}’\text{s}\;\text{accuracy}=\frac{n_{\text{kk}}}{n_{+k}}$$

(3) $$\mathrm{Overall}\mathrm{accuracy}=\frac{\sum_{k=1}^{m}n_{\text{kk}}}{n}\times 100\%$$

(4) $$Kappa\;coefficient=\frac{n\sum_{k=1}^{m}n_{\text{kk}}-\;\sum_{k=1}^{m}n_{k+}n_{+k}}{n^{2}-\;\sum_{k=1}^{m}n_{k+}n_{+k}\;}$$

where n_kk_ is an element in the k-th row and k-th column, n_k+_ is the sum of the row, n_+k_ is the sum of the column, and n is the total number of testing pixels.

## 3. Results and Discussion

**Figure 3:**
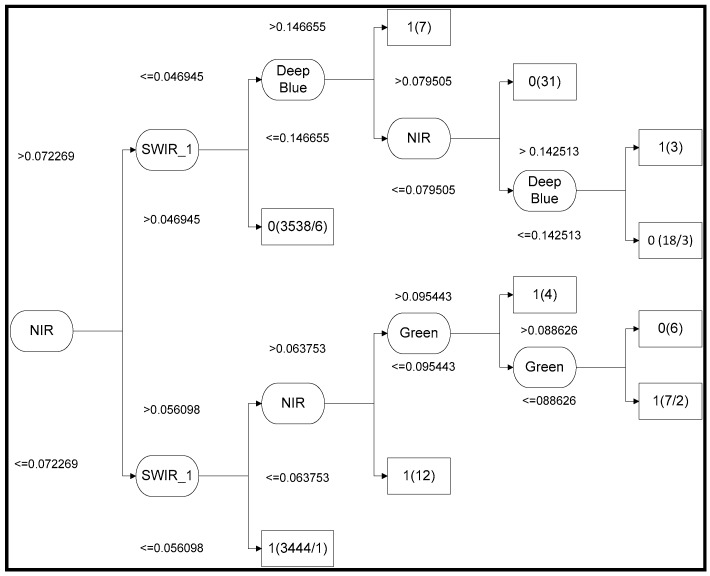
J48 decision tree model for water identification at the test site using OLI bands.

The JDT developed using the training data is shown in Figure 3. The size of the tree is 19, and it consists of 10 leaves. Figure 3 shows that the most important classification role is played by the NIR band, followed by the SWIR1, deep blue, and green bands, whereas the SWIR_2, blue, and red bands were rejected from the decision tree. NIR and SWIR can classify most of the sample points, whereas the deep blue and green bands display less significant classifications. The new additional deep blue band, which is a visible channel specifically designed for water resources and coastal zone investigation showed third highest information gain in the decision tree.

The JDT model classified 99.83% of instances correctly and had a kappa statistic of 0.9966 and area under the curve (AUC) value of 0.999. After the stratified random sampling points, additional training points were added for small water bodies, bridges, and small islands in lakes. The high classification accuracy was expected due to labelling of training samples based on known water maps and expert opinions. Hence, the data not used for training are used to evaluate the model.

**Figure 4:**
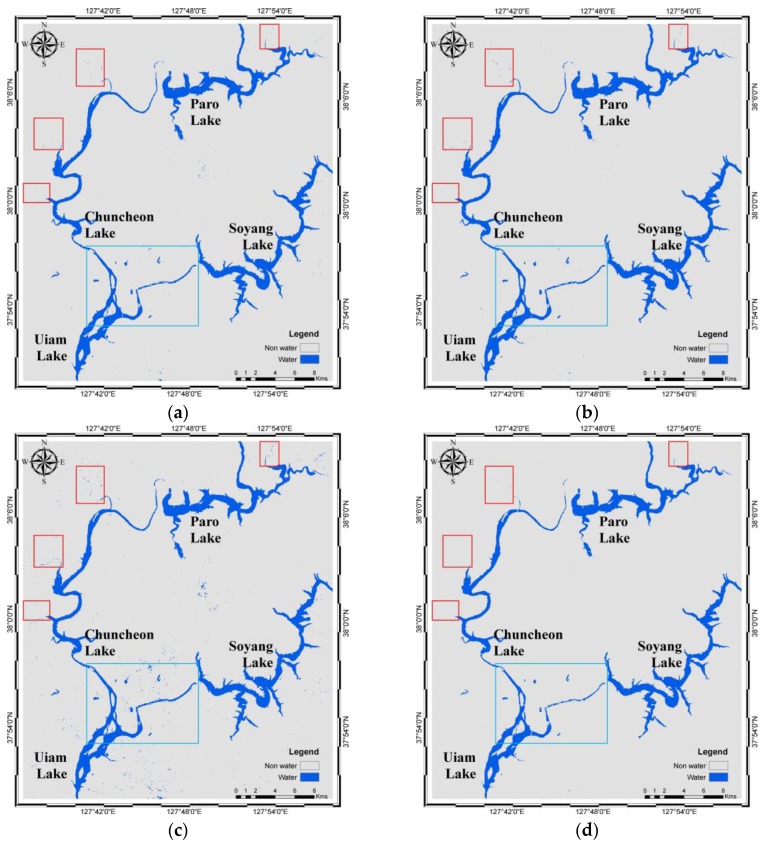

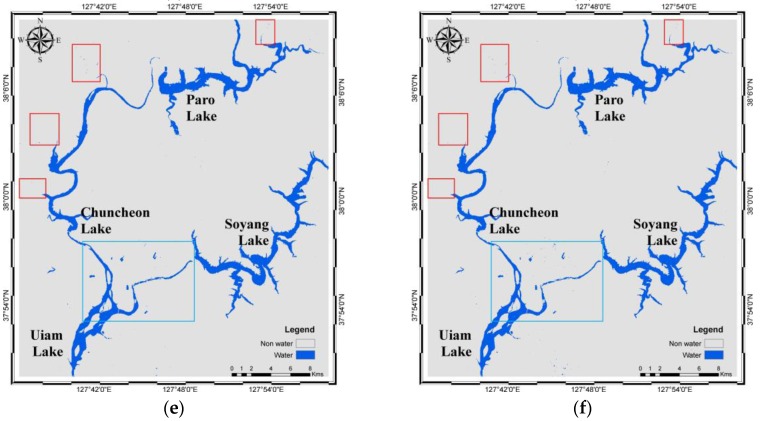
Water body identification results at the test site: (**a**) density slicing; (**b**) NDWI; (**c**) MNDWI; (**d**) ML; (**e**) SVM; and (**f**) JDT. Red boxes show smaller river network ends, whereas the light blue box inside the images shows the area with a complex urban area, smaller water bodies and a lake with bridges.

Using the model, water bodies (blue) and non-water (grey) were derived. Similarly, the results from five other methods were also derived. The results are shown in Figure 4. A total of 3065 pixels were successfully validated for water and non-water using the error matrices and their related statistics based on density slicing (Table 5), NDWI (Table 6), MNDWI (Table 7), ML (Table 8), SVM (Table 9), and JDT (Table 10). Table 10 shows that the producer’s accuracy values of water and non-water objects of the JDT are 0.9920 and 0.9909, respectively. Similarly, the water and non-water objects derived using JDT exhibit user’s accuracy values of 0.9907 and 0.9922, respectively. The overall accuracies of density slicing, NDWI, MNDWI, ML, SVM, and JDT are 99.35%, 98.92%, 98.43%, 99.28%, 99.41%, and 99.15%, respectively. Similarly, the kappa coefficients are 0.9870, 0.9785, 0.9687, 0.9856, 0.9883, and 0.9830 for density slicing, NDWI, MNDWI, ML, SVM, and JDT, respectively.

Visually, except those based on MNDWI, the results smoothly and cleanly show water and non-water objects (Figure 4). The misidentified water pixels in MNDWI are found in an agricultural area where black plastics are used for mulching potatoes, corn, and other crops. The dark plastics absorb the SWIR bands, resulting in minimal reflectance and imitating water-like characteristics. This was also the main reason for misidentification using density slicing of the SWIR_1 band and some of the JDT misidentification. Hence, seasonal variability should be very carefully noted when identifying water in agricultural areas. However, this error has been well addressed by NDWI, MLM, and SVM. Additionally, all of the methods failed to delineate water bodies with narrow widths in river networks and those covered by grasslands (red boxes in Figure 4).

**Figure 5:**
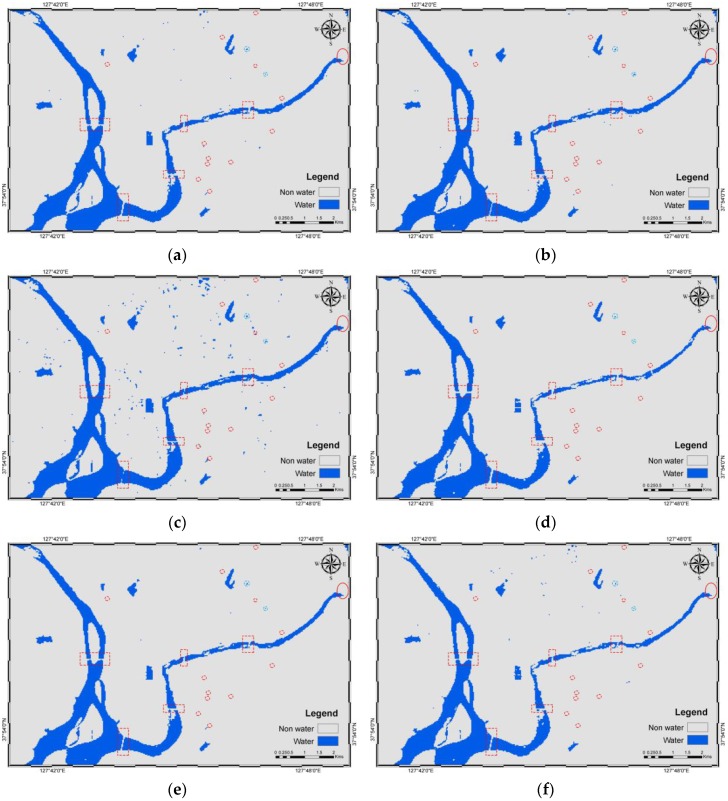
Enlarged section of the study area with smaller water bodies in a complex urban area and a lake with bridges: (**a**) Density Slicing; (**b**) NDWI; (**c**) MNDWI; (**d**) ML; (**e**) SVM; and (**f**) JDT. The red dotted boxes, red oval, red dotted circles and light blue dotted circles show bridges, a dam, unidentified water bodies and identified water bodies, respectively.

**Figure 6:**
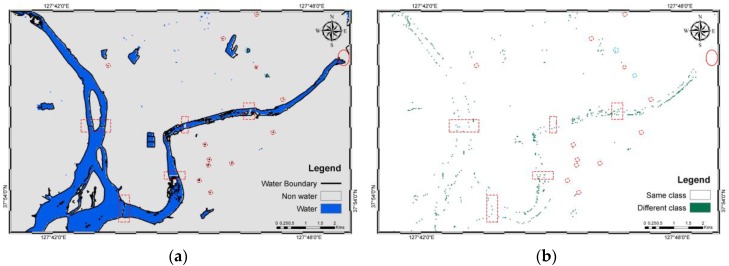

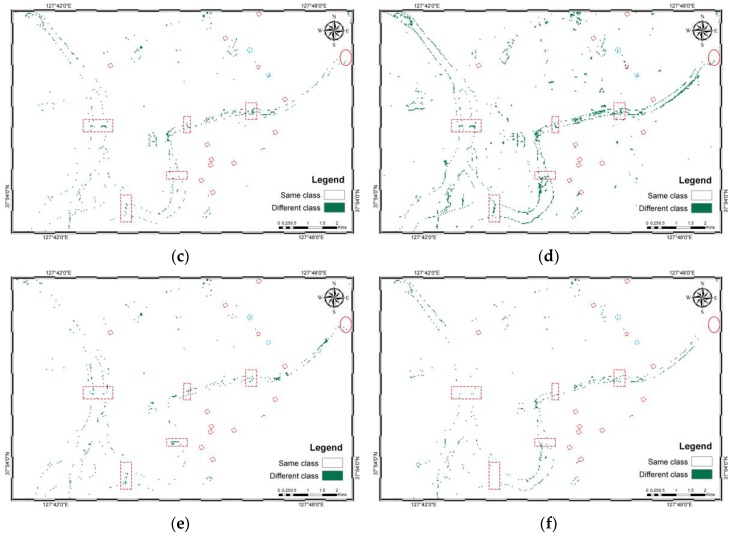
Comparison of classes derived with respect to JDT: (**a**) Water bodies identified by JDT with water boundaries extracted from digital topographic map ver. 2.0 (**b**) JDT and Density Slicing (**c**) JDT and NDWI; (**d**) JDT and MNDWI; (**e**) JDT and ML and (**f**) JDT and SVM. Similar to Figure 5, the red dotted boxes, red oval, red dotted circles and light blue dotted circles show bridges, a dam, unidentified water bodies and identified water bodies, respectively.

Figure 5 shows the central part of the study area (light blue box in Figure 4), with smaller water bodies in a complex urban area and a lake with bridges. In the figure, the red oval is an earthen dam at Soyang Lake, which is well-identified by all of the methods. Similarly, the red dotted boxes show the bridges in the figures. The bridges have been well identified and are parallel to the axes of pixels, except in the case of MNDWI, and somewhat in NDWI. The misidentification is due to the shadows of overhead structures on and under the bridge. Water bodies (light blue dotted circles) larger than 30 m × 30 m, which is the spatial resolution of OLI, were successfully identified, whereas smaller (red dotted circles) water bodies were not. The spatial resolution plays an important role in pixel-based classification of multispectral imagery, which limited the identification of smaller water bodies. The same reason caused variation in classification of water bodies at land-water boundaries. Figure 6b–f shows that the mixed pixels at the edges of water bodies are often misclassified.

NDWI and MNDWI show more disagreement compared to the other methods. From the confusion matrix statistics, NDWI, MNDWI, and SVM show the highest accuracies compared to other methods for water body identification, whereas density slicing and JDT had less misclassified non-water bodies. JDT had a similar overall accuracy and kappa coefficient as density slicing, ML, and SVM. The improvements in Landsat 8 have shown high accuracy in water identification methods, especially in density slicing of SWIR_1. A previous study conducted by Ko et al. [26] noted higher accuracy results for SVM and their proposed method, which agrees with our case study. In their study, the variables were original bands, water indices and the combination of two boosted random forest classifiers, which were used to calculate weights for water body identification. In order to further improve accuracy of the JDT in identification of water bodies, the additional explanatory variables could be introduced along with OLI bands.

## 4. Conclusions

Water is an important part of any ecosystem. Identification of water is very important for various scientific estimations, as well as social problem-solving. Many methods have been developed, and new approaches are being explored. Landsat imagery has been widely used to identify water bodies. Due to the introduction of new OLI sensors on Landsat 8, with high sensitivity associated with spectral resolution and improved signal-to-noise ratios due to the radiometric performance, the quality of imagery sensed by Landsat 8 has improved. However, according to improved quality and data size, it is necessary to explore appropriate and practical water identification methods that take advantage of improved images and minimize data inputs.

In this study, we applied and explored the effectiveness of a JDT to identify water and non-water bodies using a Landsat 8 OLI image and only its original OLI reflectance bands. Stratified randomly-sampled pixels were labelled based on a digital topographic map provided by the National Geographical Information Institute, Korea, and expert opinions to train (70%) and validate (30%) the model. The JDT model classified 99.83% of instances correctly and had kappa Statistic of 0.9966 and an AUC value of 0.999. The model was used to develop binary water and non-water maps. Similarly, water and non-water maps based on five other methods were developed and cross-compared using a confusion matrix and related statistics. The overall accuracies of density slicing, NDWI, MNDWI, ML, SVM, and JDT were 99.35%, 98.92%, 98.43%, 99.28%, 99.41%, and 99.15%, respectively. Similarly, the kappa coefficients were 0.9870, 0.9785, 0.9687, 0.9856, 0.9883, and 0.9830 for density slicing, NDWI, MNDWI, ML, SVM, and JDT, respectively. Based on these statistics and visual interpretation, almost all methods displayed high accuracies, except MNDWI, which misclassified many non-water features. Seasonal variability should be very carefully noted when identifying water in agricultural areas. Similarly, spatial resolution plays an important role in pixel-based classification of multispectral imagery, it should be carefully considered for the identification of smaller water bodies. In the current study site, using one band (density slicing) or two bands (NDWI and MNDWI) water bodies were identified with high accuracy and yet had many misidentified pixels, whereas ML and SVM also showed high accuracies but used all input bands for classification. However, JDT only used four OLI bands and had much fewer misclassified bodies. Overall, the improvement in OLI imagery shows high accuracy for various methods, including JDT, for identification of water bodies. In the decision tree, the deep blue band was found to have the third-highest information gain, which validates the importance of the band in case of water identification.

Future work regarding this water identification method will utilize data from other parts of Korea, including from complex watersheds or flooded areas, to further identify the abilities of various methods based on improved OLI imagery. New water identification methods will be used to assess and compare the accuracies, along with additional explanatory variables and additional sensor imagery. This will allow for a more comprehensive understanding of JDT classification. Analyses such as these may also be useful in other fields with binary classification problems, provided that training data are chosen carefully.

## Figures and Tables

**Table 1:** Comparing the differences between previous versions of Landsat.

Band Name	Band Number			Differences in Landsat 8
	Landsat 8	Landsat 7	Landsat 5	
Deep Blue	1	-	-	new
Blue	2	1	1	more narrow
Green	3	2	2	more narrow
Red	4	3	3	more narrow
Near Infrared (NIR)	5	4	4	more narrow
Short-wave Infrared 1 (SWIR_1)	6	5	5	more narrow
Short-wave Infrared 2 (SWIR_2)	7	7	7	more narrow
Panchromatic	8	8	-	more narrow
				only visible (red-green)
Cirrus	9	-	-	new
Long-wave Infrared (LWIR) 1	10	6	6	two bands instead of one
Long-wave Infrared (LWIR) 2	11	6	6	two bands instead of one

**Table 2:** Specifications of Landsat 8 OLI bands used.

Row/Path	Band Name	Wavelength (μm)	Resolution (m)
115/34	Deep Blue	0.435–0.451	30
	Blue	0.452–0.512	
	Green	0.533–0.590	
	Red	0.636–0.673	
	Near Infrared (NIR)	0.851–0.879	
	Short-wave Infrared 1 (SWIR_1)	1.566–1.651	
	Short-wave Infrared 2 (SWIR_2)	2.107–2.294	

**Table 3:** Multiband indexes used for water feature extraction.

Multiband Index	Equation	Remark	Reference
Normalized Difference Water Index	NDWI = (Green − NIR)/(Green + NIR)	Water has positive value	[10]
Modified Normalized Difference Water	MNDWI = (Green − SWIR_1)/(Green + SWIR_1)	Water has positive value	[12]

**Table 4:** Layout of the confusion matrix.

Classified Image	Reference Data			
	Class A	Class B	Class C	Row Total
Class A	n_AA_	n_AB_	n_AC_	n_A+_
Class B	n_BA_	n_BB_	n_BC_	n_B+_
Class C	n_CA_	n_CB_	n_CC_	n_C+_
Column Total	n_+A_	n_+B_	n_+C_	N

**Table 5:** Validation statistics of density slicing of the SWIR_1 band for water body identification.

Class	Water	Non-Water	Sum	User’s Accuracy
Water	1533	16	1549	0.98967
Non-Water	4	1533	1537	0.99740
Sum	1537	1549	3086	
Producer’s Accuracy	0.99740	0.98967		
Overall Accuracy	99.35%		Kappa coefficient	0.9870

**Table 6:** Validation statistics of NDWI for water body identification.

Class	Water	Non-Water	Sum	User’s Accuracy
Water	1516	0	1516	1.00000
Non-Water	33	1516	1549	0.97870
Sum	1549	1516	3065	
Producer’s Accuracy	0.97870	1.00000		
Overall Accuracy	98.92%		Kappa coefficient	0.9785

**Table 7:** Validation statistics of MNDWI for water body identification.

Class	Water	Non-Water	Sum	User’s Accuracy
Water	1516	0	1516	1.00000
Non-Water	48	1501	1549	0.96901
Sum	1564	1501	3065	
Producer’s Accuracy	0.96931	1.00000		
Overall Accuracy	98.43%		Kappa coefficient	0.9687

**Table 8:** Validation statistics of ML for water body identification.

Class	Water	Non-Water	Sum	User’s Accuracy
Water	1496	2	1498	0.99866
Non-Water	20	1547	1567	0.98724
Sum	1516	1549	3065	
Producer’s Accuracy	0.98681	0.99871		
Overall Accuracy	99.28%		Kappa coefficient	0.9856

**Table 9:** Validation statistics of SVM for water body identification.

Class	Water	Non-Water	Sum	User’s Accuracy
Water	1531	0	1531	1.00000
Non-Water	18	1531	1549	0.98838
Sum	1549	1531	3080	
Producer’s Accuracy	0.98838	1.00000		
Overall Accuracy	99.41%		Kappa coefficient	0.9883

**Table 10:** Validation statistics of J48 Decision Tree model for water body identification.

Class	Water	Non-Water	Sum	User’s Accuracy
Water	1504	14	1518	0.99078
Non-Water	12	1535	1547	0.99224
Sum	1516	1549	3065	
Producer’s Accuracy	0.99208	0.99096		
Overall Accuracy	99.15%		Kappa coefficient	0.9830

## Abbreviations

The following abbreviations are used in this manuscript:

AWEI	Automated Water Extraction Index
JDT	J48 Decision Tree
MNDWI	Modified Normalized Difference Water Index
NDWI	Normalized Difference Water Index
OLI	Operational Land Imager
TIRS	Thermal Infrared Sensor
